# Trace Amine-Associated Receptors as Novel Therapeutic Targets for Immunomodulatory Disorders

**DOI:** 10.3389/fphar.2018.00680

**Published:** 2018-07-02

**Authors:** Sherri L. Christian, Mark D. Berry

**Affiliations:** Department of Biochemistry, Memorial University of Newfoundland, St. John’s, NL, Canada

**Keywords:** Crohn’s disease, tyramine, phenylethylamine, inflammatory bowel disease, trace amines, leukocytes, immune system

## Abstract

Trace amines and their receptors (trace amine-associated receptors; TAARs) are an emerging pharmacological target for the treatment of human disorders. While most studies have focused on their therapeutic potential for neurologic and psychiatric disorders, TAARs are also expressed throughout the periphery, including prominent expression in human leukocytes. Furthermore, recent independent, unbiased metabolomic studies have consistently identified one or more TAAR ligands as potential etiologic factors in inflammatory bowel disease (IBD). The putative role of TAARs in diseases such as IBD that are associated with hyperactive immune responses has not, however, previously been systematically addressed. Here, we review the current state of the knowledge of the effects of TAARs on leukocyte function, in particular in the context of mucosal epithelial cells that interface with the environment; developing a model whereby TAARs may be considered as a novel therapeutic target for disorders associated with dysregulated immune responses to environmental factors. In this model, we hypothesize that altered trace amine homeostasis results in hyperactivity of the immune system. Such loss of homeostasis can occur through many different mechanisms including TAAR polymorphisms and altered trace amine load due to changes in host synthesis and/or degradative enzymes, diet, or microbial dysbiosis. The resulting alterations in TAAR functioning can then lead to a loss of homeostasis of leukocyte chemotaxis, differentiation, and activation, as well as an altered ability of members of the microbiota to adhere to and penetrate the epithelial cell layers. Such changes would generate a pro-inflammatory state at mucosal epithelial barrier layers that can manifest as clinical symptomatology such as that seen in IBD. These alterations may also have the potential to induce systemic effects, which could possibly contribute to immunomodulatory disorders in other systems, including neurological diseases.

## Introduction

Since the discovery of trace amine-associated receptors (TAARs) in 2001 (Borowsky et al., 2001; Bunzow et al., 2001), there has been a gradually growing interest in the therapeutic potential of this family of receptors. This interest has largely focused on the most well-characterized family member, TAAR1, and its potential as a novel target for pharmacotherapy of psychiatric disorders, in particular schizophrenia (Revel et al., 2013) and drug abuse (Pei et al., 2016), and more recently metabolic disorders (Raab et al., 2016). In this article, we will present evidence that leads us to propose that TAARs also comprise a potential therapeutic target for disorders associated with an environmentally induced hyper-reactive immune state. Recent review articles have thoroughly covered general (Pei et al., 2016) and human (Berry et al., 2017) TAAR pharmacology to which the reader is referred for more detailed background information. Here, we will focus our discussion on recent evidence that supports a role for TAARs in regulating immune cell functions, and also implicates changes in endogenous TAAR ligands in the etiology of dysfunctional immune responses at epithelial cell barrier layers, with a particular view toward the gastrointestinal tract, which has had the most focus up until now. From these studies, we will develop the hypothesis that TAARs are novel therapeutic targets for environmental hypersensitivity and other immunomodulatory disorders.

## Evolution and Genetics of Human TAARs

Compared to many other G protein-coupled receptors, TAARs show a quite pronounced species-dependent diversity due to a large number of species-specific duplications, deletions, and pseudogenizations. In mammals, each species’ TAAR complement clusters together as single exon genes (with the exception of TAAR2 which contains two exons) on a single chromosome. Within the nine mammalian TAAR sub-families, there are two distinct clades comprising TAAR1–4 and TAAR5–9. TAAR1–4 are evolutionarily the oldest members, with TAAR1 the oldest and TAAR4 the second oldest (Lindemann et al., 2005; Eyun et al., 2016), appear to be tuned to the detection of primary amines (Ferrero et al., 2012), and are under negative selection pressures (Eyun et al., 2016). By contrast, TAAR5–9 are tuned toward tertiary amines (Ferrero et al., 2012) and are under strong positive selection pressures (Hussain et al., 2009; Vallender et al., 2010), with a variety of species-specific isoforms identified (Eyun et al., 2016). Such positive selection pressures may indicate an evolutionary role in environmental adaptation responses, and indeed the TAAR expression profiles of at least some species may respond to habitat changes (Churcher et al., 2015; Fatsini et al., 2016). Furthermore, with the notable exception of TAAR1, TAARs have also now been confirmed as a new family of receptors involved in olfaction, which respond to volatile signaling molecules associated with diverse ecological contexts (Liberles, 2015).

### Human TAAR Isoforms

In humans, TAAR genes are located on chromosome 6q23.2 (Borowsky et al., 2001; Bunzow et al., 2001) with six functional genes (TAAR1, TAAR2, TAAR5, TAAR6, TAAR8, and TAAR9) and three pseudogenes (TAAR3, TAAR4, and TAAR7) present (Lindemann et al., 2005). Function-modifying polymorphisms of TAAR1 (Shi et al., 2016), TAAR2 (Bly, 2005), TAAR6 (Duan et al., 2004; Pae et al., 2008, 2010; Chang et al., 2015), and TAAR9 (Vanti et al., 2003; Muller et al., 2010) have been reported. While such genetic variants have been suggested to have clinical relevance, reports have rarely been independently validated, and studies are often confined to rather small sample sizes, making firm conclusions of the putative clinical relevance difficult.

### Endogenous TAAR Ligands

Although only TAAR1 has been officially de-orphanized (Alexander et al., 2017), a number of endogenous ligands for individual TAAR isoforms have been identified, many of which are simply the decarboxylation products of amino acids. Notable endogenous ligands of individual TAARs are 2-phenylethylamine (PEA), *p*-tyramine (TYR), and tryptamine, all of which activate TAAR1, and probably also TAAR4; trimethylamine (TMA), a tertiary amine product of the microbial degradation of carnitine and choline, is a high affinity agonist at TAAR5; and as yet unidentified TAARs are activated by the diamines putrescine and cadaverine.

#### 2-PEA and *p*-TYR

**FIGURE 1:**
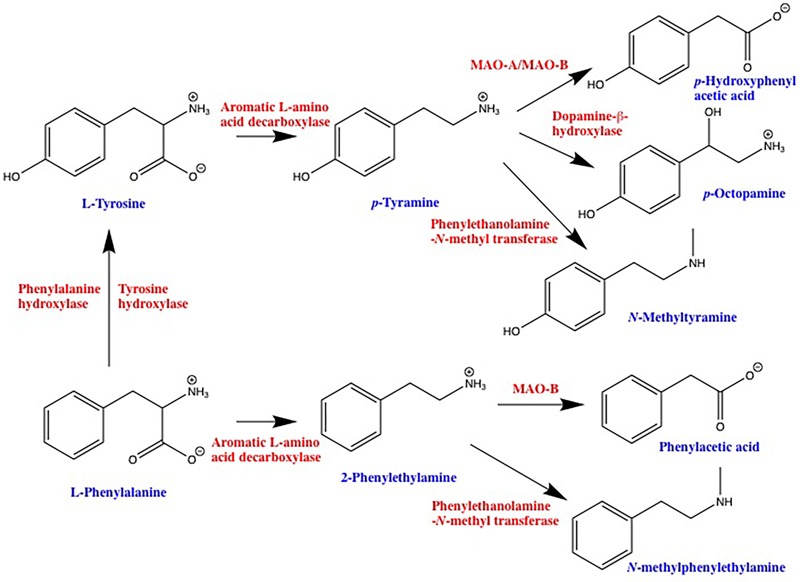
Schematic representation of trace amine synthetic and degradative pathways. 2-phenylethylamine and *p*-tyramine are primarily synthesized by decarboxylation of precursor amino acids *via* the enzyme aromatic L-amino acid decarboxylase. Depending on the cell type, L-phenylalanine can be converted into L-tyrosine *via* either tyrosine hydroxylase or a dedicated phenylalanine hydroxylase. Degradation of 2-phenylethylamine and *p*-tyramine is primarily *via* monoamine oxidase, 2-phenylethylamine being a highly selective substrate for the B isoform, whereas *p*-tyramine is a mixed monoamine oxidase-A and -B substrate. Both 2-phenylethylamine and *p*-tyramine can also be converted to a variety of other compounds that are also agonists at one or more TAAR. MAO, monoamine oxidase.

2-Phenylethylamine and *p*-TYR (Simmler et al., 2016), as well as a number of other endogenous amines such as tryptamine (Simmler et al., 2016), 3-iodothyronamine (Scanlan et al., 2004), and 3-methoxytyramine (Bunzow et al., 2001; Sotnikova et al., 2010) are selective agonists at human TAAR1. PEA and TYR are the products of aromatic L-amino acid decarboxylase (AADC)-mediated decarboxylation of L-phenylalanine and L-tyrosine, respectively, with degradation occurring primarily *via* monoamine oxidase (**Figure 1**). Subsequent enzymatic conversion to a variety of other TAAR ligands such as *p*-octopamine, *N*-methylphenylethylamine, and *N*-methyltyramine is also possible (**Figure 1**). While PEA synthesis is often reported as being neuronal, AADC is also present in a number of non-neuronal tissues including epithelial cells of the gastrointestinal tract (Lauweryns and Van Ranst, 1988; Vieira-Coelho and Soares-da-Silva, 1993) and lungs (Lauweryns and Van Ranst, 1988; Linnoila et al., 1993). In addition, L-phenylalanine and L-tyrosine are readily decarboxylated by commensal prokaryotes (Marcobal et al., 2006; Irsfeld et al., 2013; Williams et al., 2014) providing an additional pathway for the production of PEA and TYR in tissues that interface with the environment. Furthermore, dietary intake provides a third source of PEA and TYR in the gastrointestinal tract. Both compounds have long been recognized as being enriched in commonly ingested foods; with aged cheeses, red wine, chocolate, and fermented meats particularly well documented for their enriched levels of PEA and/or TYR (Coutts et al., 1986). In general, any foods that involve anaerobic fermentation as part of their production can be expected to contain elevated levels of one or more trace amines (Toro-Funes et al., 2015; Gardini et al., 2016; Pessione and Cirrincione, 2016). Indeed, the ability to detect increased levels of compounds such as TYR and PEA in microbe-contaminated foods may be one of the evolutionary driving forces for the appearance of TAARs in the olfactory epithelium. Seafood also provides a rich source of TYR and PEA, as well as other putative TAAR ligands (An et al., 2015; Biji et al., 2016). The potential of such dietary sources to lead to the activation of host TAARs, particularly in sensitive individuals, has only just begun to be recognized by those with research interests other than TAARs (Ohta et al., 2017). All known endogenous ligands for TAARs are volatile, and have been identified in the urine and feces from a number of species. As such, inhalation of TAAR ligands associated with environmental sources is an additional source of trace amines in the pulmonary system.

Once synthesized, ingested, or inhaled, there appears to be no storage mechanism for either PEA or TYR in mammalian cells. The half-life for the endogenous pool is less than 30 s (Durden and Philips, 1980) indicating a very high turn-over rate with typical tissue concentrations being in the 1–10 ng/g tissue range, equating to approximately 10–100 nM (Berry, 2004). Release of TYR and PEA from neuronal preparations is dependent solely on the total tissue levels (Henry et al., 1988; Dyck, 1989), consistent with a diffusion-mediated process and a lack of storage (Juorio et al., 1988). We have confirmed ready diffusion of TYR across synthetic lipid bilayers (Berry et al., 2013), and in native membrane preparations this appears to be further enhanced by the presence of organic cation transporter 2 (OCT2; *Slc22A2*), a bidirectional facilitated diffusion transporter which exhibits a *K*_t_ for TYR of ≃100 nM (Berry et al., 2016). Taken together, these studies indicate that PEA and TYR readily cross cell membranes, a situation that means local levels of PEA and TYR exist in a steady state and are sensitive to fluctuations in synthesis and/or degradation.

#### Trimethylamine

During the initial classification of TAARs as receptors involved in olfaction, TMA was identified as a potent, selective agonist at human TAAR5 (Liberles and Buck, 2006), a finding that has subsequently been independently verified (Wallrabenstein et al., 2013; Zhang et al., 2013). Interestingly, there is no known mammalian pathway for the production of TMA. Rather, TMA synthesis appears to be due to the action of the constitutive microbiota on betaine, carnitine, and (phosphatidyl)choline (Craciun and Balskus, 2012; Zhu et al., 2014; Kalnins et al., 2015). As such, TMA represents a good candidate molecule for involvement in microbe-host signaling cascades. Moreover, elevated TMA levels have been found to result from dysbiosis in the intestines, vagina, and oral cavity (Fennema et al., 2016; Zhang and Davies, 2016). Degradation of TMA occurs almost exclusively in the liver through the action of flavin monooxygenase 3 (FMO3) (Fennema et al., 2016). This raises interesting pharmacokinetic questions since the site of degradation is relatively distant from the site of production, thus raising the possibility of tonic activation of any TAAR5 that is present between the two sites. Indeed, TMA has been identified as a constituent of numerous body fluids (Mitchell and Smith, 2016). This possibility is further strengthened when considering that the *K*_m_ of FMO3 for TMA is 28 μM (Lang et al., 1998), 100-fold in excess of the EC_50_ of TMA at human TAAR5 (Berry et al., 2017). Outside of the mammalian body, TMA production is associated with bacterial degradation of meat products, in particular fish (Horowitz et al., 2014).

#### Cadaverine, Putrescine, and Other Polyamines

In experimental animals, the pan knockout of the TAAR2–TAAR9 cluster eliminates behavioral responses to the presence of cadaverine and putrescine (Dewan et al., 2013). Furthermore, a number of teleost-specific TAAR isoforms are also activated by cadaverine (Hussain et al., 2013) and other diamines (Li et al., 2015). The relevance of these observations to human physiology is supported by the recent identification of a putative diamine-binding site in human TAAR6 and TAAR8 (Li et al., 2015; Izquierdo et al., 2018), which has been predicted *via in silico* analysis to bind cadaverine and putrescine (Izquierdo et al., 2018).

Both putrescine and cadaverine are produced by the decarboxylation of precursor L-amino acids, ornithine *via* ornithine decarboxylase in the case of putrescine, and lysine *via* lysine decarboxylase in the case of cadaverine (Miller-Fleming et al., 2015; Pegg, 2016). Like other TAAR ligands, both are also produced by microbial action on proteinaceous materials, and as such are associated with decaying flesh. Indeed, putrescine and cadaverine are often credited with being the “smell of death.”

### Tissue and Cellular Distribution of TAARs

One of the factors that has hampered the advancement of TAAR pharmacology has been a general lack of suitable reagents to probe the individual isoforms (see Berry et al., 2017 for details). Basal expression levels of TAARs are generally very low, and only TAAR1 has been studied in detail. Indeed, TAAR1 is the only isoform for which a well-validated anti-human antibody has been described (Raab et al., 2016). With respect to the TAAR family in general, the most well-replicated finding has been the presence of all TAAR isoforms except TAAR1 in the olfactory epithelium, including in humans (Liberles and Buck, 2006; Hashiguchi and Nishida, 2007; Gliem et al., 2009; Horowitz et al., 2014; Kanageswaran et al., 2015; Syed et al., 2015). In these olfactory systems, individual isoforms have been shown to respond to a variety of volatile amines associated with distinct ecological cues (Ferrero et al., 2011; Dewan et al., 2013; Li et al., 2013; Zhang et al., 2013; Liberles, 2015; Santos et al., 2016) including predator presence, spoiled food, and conspecifics of the opposite gender. In addition, individual TAARs are also expressed throughout the body, including both central nervous and peripheral organ systems (Berry et al., 2017).

#### TAAR1

While TAAR1 is well established as being expressed throughout the brain of experimental animals, primarily associated with the major monoaminergic (in particular, although not exclusively, dopaminergic) nuclei and projection areas, few studies have attempted to confirm such findings in humans (Borowsky et al., 2001; Lindemann et al., 2005). It is likely that a similar distribution is present in humans, a situation supported by the observation of a normalization of functional magnetic resonance imaging (fMRI) in various brain regions, in particular those associated with the dopaminergic reward circuitry, by a mixed 5-HT_1A_/TAAR1 compound in patients with schizophrenia (Nazimek et al., 2016). Similar modifications of brain activity in response to a highly selective TAAR1 agonist had previously been reported using pharmacological MRI in rodent models (Revel et al., 2013). Expression of TAAR1 has also been reported to be present in the spinal cord of multiple species including humans (Borowsky et al., 2001; Lindemann et al., 2008; Gozal et al., 2014), as well as in human astrocytes (Cisneros and Ghorpade, 2014).

TAAR1 expression is also present in a number of nutrient sensing organs in humans, where it appears to regulate hormone secretion in response to nutrients (Adriaenssens et al., 2015; Raab et al., 2016). Specifically, TAAR1 has been confirmed to be present in pancreatic β-cells (Revel et al., 2013; Raab et al., 2016), D cells of the stomach (Adriaenssens et al., 2015; Raab et al., 2016), and enterochromaffin mucosal cells of the intestines (Kidd et al., 2008; Raab et al., 2016). Expression in human leukocytes (D’Andrea et al., 2003), including peripheral mononuclear (PMN) cells (Nelson et al., 2007), B lymphocytes (Wasik et al., 2012; Babusyte et al., 2013), monocytes (Babusyte et al., 2013), polymorphonuclear leukocytes (Babusyte et al., 2013), NK cells (Babusyte et al., 2013), and T lymphocytes (Babusyte et al., 2013; Sriram et al., 2016), has also been established (**Table 1**). Furthermore, methamphetamine-mediated stimulation of T lymphocytes has been reported to increase TAAR1 expression levels (Sriram et al., 2016). Given that methamphetamine itself is a TAAR1 agonist (Bunzow et al., 2001; Simmler et al., 2016), this raises the possibility of a positive feedback, amplification loop for TAAR1 signaling in T lymphocytes. Such a positive feedback loop has recently been described in response to the highly selective TAAR1 agonist RO5203648 in placental cells (Stavrou et al., 2018).

**Table 1:** TAAR expression in human leukocyte populations.

Receptor isoform	Immune cell type	Transcript or protein
TAAR1	Unspecified leukocytes	mRNA (D’Andrea et al., 2003)
	Peripheral mononuclear cells	mRNA (Nelson et al., 2007)
	B cells	mRNA and protein (Babusyte et al., 2013; Sriram et al., 2016)
	Monocytes	mRNA (Babusyte et al., 2013)
	PML	mRNA and protein (Babusyte et al., 2013)
	NK cells	mRNA (Babusyte et al., 2013)
	T cells	mRNA and protein (Babusyte et al., 2013; Sriram et al., 2016)
TAAR2	Peripheral mononuclear cells	mRNA (Nelson et al., 2007)
	B cells	mRNA (Nelson et al., 2007; Babusyte et al., 2013)
	Monocytes	mRNA (Babusyte et al., 2013)
	PML	mRNA and protein (Babusyte et al., 2013)
	NK cells	mRNA (Babusyte et al., 2013)
	T cells	mRNA (Babusyte et al., 2013)
TAAR5	Not present (Nelson et al., 2007)	
	B cells	mRNA (Babusyte et al., 2013)
	Monocytes	mRNA (Babusyte et al., 2013)
	PML	mRNA (Babusyte et al., 2013)
	NK cells	mRNA (Babusyte et al., 2013)
	T cells	mRNA (Babusyte et al., 2013)
TAAR6	Unspecified leukocytes	mRNA (D’Andrea et al., 2003)
	B cells	mRNA (Babusyte et al., 2013)
	Monocytes	mRNA (Babusyte et al., 2013)
	PML	mRNA (Babusyte et al., 2013)
	NK cells	mRNA (Babusyte et al., 2013)
	T cells	mRNA (Babusyte et al., 2013)
TAAR8	Unspecified leukocytes	mRNA (D’Andrea et al., 2003)
	Not present (Babusyte et al., 2013)	
TAAR9	Unspecified leukocytes	mRNA (D’Andrea et al., 2003)
	B cells	mRNA (Babusyte et al., 2013)
	Monocytes	mRNA (Babusyte et al., 2013)
	PML	mRNA (Babusyte et al., 2013)
	NK cells	mRNA (Babusyte et al., 2013)
	T cells	mRNA (Babusyte et al., 2013)

Notably, the available evidence indicates that TAAR1 resides primarily intracellularly in all tissues and cell types studied (Lindemann et al., 2005; Barak et al., 2008; Pei et al., 2016; Raab et al., 2016), only translocating to the plasma membrane following agonist-induced receptor heterodimerization (Espinoza et al., 2011, 2015; Harmeier et al., 2015). Therefore, the ability of PEA and TYR to readily cross cell membranes either by diffusion (Berry et al., 2013), OCT2-mediated transport (Berry et al., 2016), or *via* other as yet unidentified transporters, is a critical first step in TAAR1 signaling cascades.

#### TAAR2

TAAR2 is a little unusual, being the only family member with a gene containing more than one exon (Lindemann et al., 2005). Very little is known, however, about its distribution. In human leukocytes, there is some evidence that TAAR2 expression may mirror that of TAAR1, with TAAR2 mRNA reported in B lymphocytes, monocytes, NK cells, and T lymphocytes (Nelson et al., 2007; Babusyte et al., 2013), and both mRNA and protein reported in granulocytes (Babusyte et al., 2013). The co-localization of TAAR1 and TAAR2 may be functionally important, with heterodimerization between the two having possible consequences to TAAR1 signaling (Babusyte et al., 2013). In laboratory rodents, TAAR2 mRNA has also been reported in duodenal mucosal cells (Ito et al., 2009), heart (Chiellini et al., 2007), and testis (Chiellini et al., 2012).

#### TAAR5

Although there is a known ligand (TMA) identified that is well established to be present in humans, very few studies have investigated the expression profile of TAAR5. A potential preferential expression in human B lymphocytes has been suggested, with low level expression in other leukocyte populations (Babusyte et al., 2013). In rodents, TAAR5 mRNA is expressed in brain, with a distribution partially overlapping that of TAAR1 (Dinter et al., 2015). Spinal cord (Gozal et al., 2014) and testis (Chiellini et al., 2012) expression has also been suggested.

#### TAAR6

In human brain, TAAR6 transcript levels may surpass those of TAAR1 (Duan et al., 2004), and also be primarily localized to known monoaminergic projection pathways including amygdala (Borowsky et al., 2001; Duan et al., 2004), basal ganglia (Duan et al., 2004), frontal cortex (Duan et al., 2004), hippocampus (Borowsky et al., 2001; Duan et al., 2004), and substantia nigra (Duan et al., 2004). Outside of the brain, TAAR6 transcripts have also been reported in kidney (Borowsky et al., 2001), and leukocytes (D’Andrea et al., 2003; Babusyte et al., 2013). Although present in rat spinal cord (Gozal et al., 2014), no transcripts were found in human (Duan et al., 2004).

#### TAAR8

Astroglial TAAR8 transcripts have been reported to be increased following activation by lipopolysaccharide treatment (D’Andrea et al., 2012). Elsewhere in the human brain, TAAR8 mRNA was reported to be present in the amygdala (Borowsky et al., 2001). D’Andrea et al. (2003) also reported TAAR8 transcripts in leukocytes, although this was not confirmed by others (Babusyte et al., 2013). Whether lipopolysaccharide activation of leukocytes increases TAAR8 levels as observed in astrocytes is unknown. Expression of TAAR8 in the kidney has also been suggested (Borowsky et al., 2001).

#### TAAR9

All leukocyte populations studied have been reported to express TAAR9 (D’Andrea et al., 2003; Babusyte et al., 2013), with human expression also suggested in the spleen (Regard et al., 2008), pituitary gland (Vanti et al., 2003), and skeletal muscle (Vanti et al., 2003).

## TAARs and the Immune System

Overall, the evidence described above indicates that multiple TAARs are expressed in the immune system, in particular in various populations of leukocytes (**Table 1**), and that in at least some cases, this is regulated in response to leukocyte activation. This raises the possibility that TAARs and their ligands may comprise a hitherto unsuspected target for modulation of immune function. Furthermore, given the ready production of such ligands by members of the constitutive microbiota, a role for TAARs in microbiota-immune system signaling should be considered. In the following sections, we will review the current state of the knowledge of the effects of TAARs on leukocyte function, developing a model whereby TAARs are considered as potential therapeutically relevant targets in disorders associated with dysregulated immune responses to environmental factors. In particular growing evidence indicates TAARs may be attractive novel molecular targets in inflammatory bowel disease (IBD).

### TAARs Regulation of Leukocyte Function

As described above, TAARs are differentially expressed between various leukocyte populations in humans. Notably, both TAAR1 and TAAR2 expression are increased following leukocyte activation (Nelson et al., 2007; Wasik et al., 2012). The endogenous TAAR1 ligands PEA and TYR are chemotactic for PMN (Babusyte et al., 2013), and can be released from activated platelets (D’Andrea et al., 2003). It is not known if TAAR1 ligands are chemotactic for other leukocyte populations and this is an area for future study. This chemotactic response is dependent on the expression of both TAAR1 and TAAR2, possibly due to agonist-induced heterodimerization (Babusyte et al., 2013), similar to that also reported between TAAR1 and D_2_-like dopamine receptors (D2R) (Espinoza et al., 2015; Harmeier et al., 2015).

Activation of leukocyte TAAR1 may also regulate T helper (Th) cell differentiation, directing this away from the Th1 phenotype and toward the Th2 phenotype. Supporting this, a TAAR1-dependent increase in interleukin (IL)-4 secretion, decrease in IL-2 secretion, and decrease in expression of secreted phosphoprotein 1 have been reported (Babusyte et al., 2013). Promotion of the Th2 phenotype would be expected to lead to increased activation of B lymphocytes, and TAAR1 agonists do increase B cell secretion of immunoglobulin E (IgE) (Babusyte et al., 2013). TAAR1-mediated B lymphocyte apoptosis has also been reported (Wasik et al., 2012). Importantly, all of the above effects of TAAR1 agonists occur with low nanomolar EC_50_ values, well within the range of endogenous TA plasma levels (D’Andrea et al., 2003). In healthy individuals, PEA and TYR levels are tightly controlled. As previously described the endogenous pool exists in a steady state, with no storage and a half-life of less than 30 s (Durden and Philips, 1980). As such, it is possible that alterations in TAAR1 signaling can occur simply due to fluctuations in PEA and/or TYR levels, and such altered signaling could affect homeostatic immune responses.

In recent years, it has become increasingly apparent that leukocyte populations are also regulated by receptors commonly associated with neurotransmission (Pacheco et al., 2014). In particular, dopamine and its receptors have been shown to be present in various populations including B lymphocytes, dendritic cells, neutrophils, NK cells, and T lymphocytes (Pacheco et al., 2014), and to play a role in signaling between immune cells (Pacheco et al., 2014; Papa et al., 2017). Activation of individual dopamine receptors has been shown to be involved in both B lymphocyte (Papa et al., 2017) and T lymphocyte activation (Pacheco et al., 2014), T lymphocyte cytokine secretion and migration (Pacheco et al., 2014), and dependent on the dopamine receptor sub-type activated promotion of Th1, Th2, or Th17 differentiation (Pacheco et al., 2014). Since TAAR1 has been established as an endogenous modulator of D2R, TAAR-mediated regulation of dopaminergic signaling within immune cells may also occur, and is perhaps likely. In particular, TAAR1 appears to function as an endogenous rheostat, maintaining homeostasis of dopaminergic systems (Bradaia et al., 2009; Revel et al., 2013) by selectively heterodimerizing with D2R (Espinoza et al., 2011, 2015; Salahpour et al., 2012; Harmeier et al., 2015). In the central nervous system, this TAAR1 agonist-induced heterodimerization causes biased signaling involving the G protein-independent β-arrestin cascade (Espinoza et al., 2015; Harmeier et al., 2015). Intriguingly, β-arrestin isoforms have been implicated in autoimmune disorders due to regulation of both innate and adaptive immune system functions, including chemotaxis (Jiang et al., 2013), gating cytokine receptor responsitivity (Gurevich and Gurevich, 2014), Th cell differentiation (Jiang et al., 2013), epithelial cell barrier functions (Jiang et al., 2013), and inflammation-induced epithelial cell apoptosis (Zeng et al., 2015). Therefore, the potential of TAAR1 to regulate D2R signaling *via* the β-arrestin cascade in leukocytes warrants parallel systematic investigation alongside the effects of TAAR1 agonists alone.

### TAARs and the Major Histocompatibility Complex

The major histocompatibility complex (MHC) is a critical mediator of T lymphocyte activation. Degradation and presentation of antigens on either class I or class II MHC by antigen-presenting cells, such as dendritic cells or macrophages, is essential for activation of CD8+ and CD4+ T cells, respectively. Interaction of CD8+ T lymphocytes with the peptide–MHC-I complex, along with the involvement of Th cells, causes these cells to differentiate into cytotoxic T lymphocytes (CTL) that can induce the lysis of infected or altered cells. Interaction of CD4+ Th cells with the peptide–MHC-II complex induces their activation. In addition, the context provided by cytokines in the extracellular environment following release from damaged, infected, or otherwise altered cells present in the microenvironment further influences the specific differentiation of CD4+ T cells into the various Th subsets including Th1, Th2, Th17, and Treg. Intriguingly, TAARs were recently implicated in the recognition of class I MHC, at least in the context of MHC-mediated mate selection (Santos et al., 2016). Here, the TAAR3 genotype was shown to play a role in female selection of MHC-diverse males by the greater sac-winged bat. While the context of this detection is not immune cell responsitivity, and TAAR3 is a pseudogene in humans, this does indicate that the possibility of TAARs involvement in MHC-mediated responses within leukocytes should be considered, and warrants further study.

### TAARs and Their Ligands in IBD

Inflammatory bowel disease comprises a group of chronic inflammatory disorders, where Crohn’s disease (CD) and ulcerative colitis (UC) are the two major forms. The etiology of IBD involves complex multifactorial components including genetic, immunologic, and environmental influences (Kostic et al., 2014; Lane et al., 2017; Zhang M. et al., 2017) that remain to be fully elucidated. Key components of the immune response in IBD include increased leukocyte recruitment to the gastrointestinal mucosa (Vitale et al., 2017) involving both innate and adaptive immune systems, and loss of homeostatic control of Th cell differentiation into Th1 (Frank et al., 2007; Pacheco et al., 2014; Zhang M. et al., 2017), Th17 (Lee and Mazmanian, 2010; Pacheco et al., 2014; Zhang M. et al., 2017), and Treg (Pacheco et al., 2014; Zhang M. et al., 2017) phenotypes. Normalization of epithelial cell–immune system communication (Jostins et al., 2012; Martini et al., 2017) and identification of molecules that mediate leukocyte chemotaxis (Thomas and Baumgart, 2012; Bamias et al., 2013) are regarded as promising therapeutic targets. As described above, TAAR1-mediated signaling, either direct or through modulation of D2R, may regulate all these responses. Furthermore, microbial production of trace amines is also implicated in the ability of microbes to adhere to epithelial cells, and in subsequent internalization of microbes and epithelial cell cytokine production (Fernandez de Palencia et al., 2011; Luqman et al., 2018).

**FIGURE 2:**
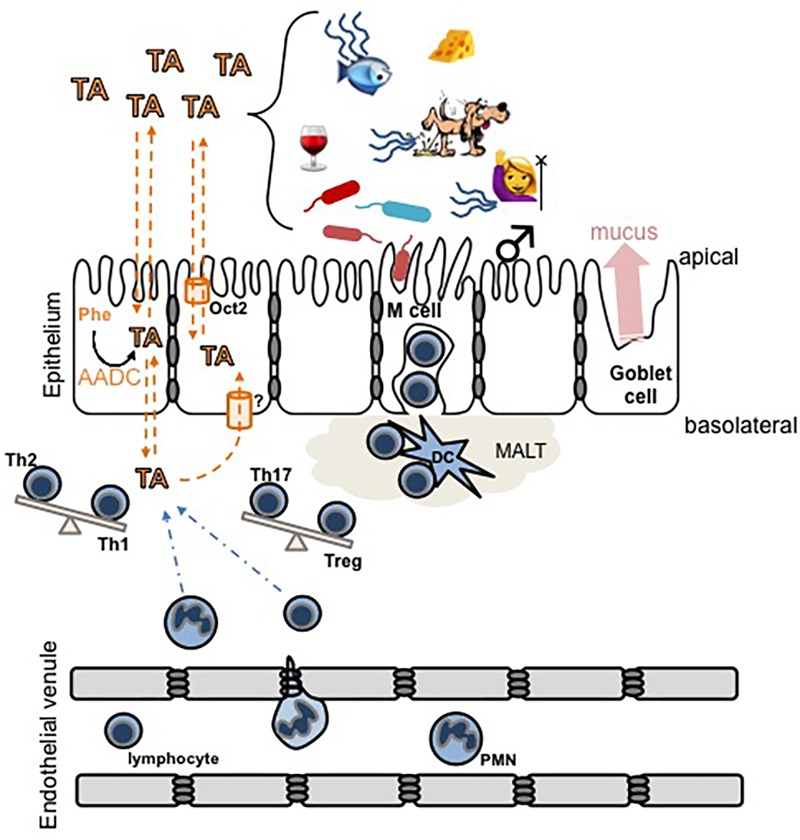
Schematic diagram of hypothetical interactions between trace amines (TA) and the mucosal epithelial layer immune system. TA levels can increase due to an increased activity of aromatic L-amino acid decarboxylase (AADC) in intestinal epithelial cells, or by exogenous sources including food, microbial activity, decaying animals, or in secretions such as urine, sweat, or breath. In humans, trimethylaminuria [fish (mal)odor syndrome] increases the secretion of TMA. TA cross lipid bilayers, including both the apical and basolateral surfaces of the intestinal epithelium, by a combination of simple diffusion and transporter-mediated facilitated diffusion. The overarching hypothesis illustrated is that pathological accumulation of TA in the basolateral compartment may (a) recruit immune cells to the area and/or (b) induce the differentiation of T cells to promote inflammation and subsequent damage to the epithelium. Dashed orange lines indicate movement of TA, dashed blue lines indicate movement of immune cells, and the balance beams indicate a potential TA-induced shift in the balance of T helper cell phenotypes. MALT, mucosal-associated lymphoid tissue; DC, dendritic cell.

Among the most prominent environmental influences in IBD are a decreased complexity of the constitutive microbiota (Frank et al., 2007; Kostic et al., 2014; Lane et al., 2017) and various dietary factors (Kostic et al., 2014; Lewis et al., 2015; Lane et al., 2017; Sigall-Boneh et al., 2017; Zhang M. et al., 2017). The presence of TAARs in almost all leukocyte populations, and the availability of their ligands from intestinal epithelial cells, the microbiota, and food, indicates that the trace aminergic system is conveniently positioned at the interface of genetics, leukocyte chemotaxis and activation, microbiota, and diet (**Figure 2**). The putative involvement of TAARs in IBD, however, has not previously been systematically addressed or formally hypothesized.

Changes in trace amine metabolism have recently been correlated to both IBD disease status (Wilson et al., 2015; Santoru et al., 2017) and the decreased microbiota complexity that is a defining characteristic of IBD (Santoru et al., 2017). Unbiased metabolomic screens have directly implicated dysregulation of known TAAR ligands in the pathology of IBD, although the presence of high affinity receptors for these compounds has been overlooked. Most notably, PEA has been reported to be increased in fecal samples from CD patients, and to be one of the metabolites that allows discrimination of CD from healthy patient populations (Jacobs et al., 2016; Santoru et al., 2017). Such results are also consistent with previous studies identifying altered tyrosine and phenylalanine metabolism as a factor for discriminating IBD from healthy populations (Burczynski et al., 2006; Jansson et al., 2009). Putrescine and cadaverine have also been identified as selectively elevated in CD patients (Santoru et al., 2017) consistent with previous reports of altered lysine metabolism (Marchesi et al., 2007). Likewise, in UC patients, significant elevations in TYR and cadaverine have been reported (Santoru et al., 2017). Furthermore, elevated levels of fecal TMA have been reported in IBD (Marchesi et al., 2007), along with both elevations and reductions of its primary metabolite trimethylamine-*N*-oxide (TMAO) (Wilson et al., 2015; Santoru et al., 2017).

Despite these identified changes in TAAR ligands, there appears to be only a single study that has identified possible roles of TAARs in IBD (Taquet et al., 2012). Here, in an unbiased microarray screen, TAAR2, TAAR5, and TAAR9 were found to be among the most highly upregulated genes in biopsies from inflamed regions of CD patients, and contributed to the separation of CD patients from those with irritable bowel syndrome (Taquet et al., 2012). Although neither PEA nor TYR are agonists at TAAR2, the aforementioned heterodimerization of TAAR2 with TAAR1 could provide a link between the altered TYR and PEA metabolomic profiles and TAAR2 over-expression, both of which allow discrimination of IBD populations. It is also worth noting that activity-modifying polymorphisms of D2R have been implicated in CD (Magro et al., 2006). As such, given the well-documented role of TAAR1 in normalizing D2R signaling, even in the absence of *de facto* changes in TAAR1 or its endogenous ligands, it may still be a viable target for pharmacotherapy. Importantly in this aspect, TAAR1-directed therapeutics have been confirmed in phase I clinical trials to be generally safe (Fowler et al., 2015).

### A TAAR-centric Hypothesis of IBD

Based on the above evidence, we hypothesize that altered trace amine homeostasis can result in hyperactivity of the immune system which clinically can manifest as IBD. Such loss of homeostasis can occur through many different mechanisms. For example: polymorphisms of one or more TAAR resulting in altered leukocyte homeostasis; changes to trace amine levels as a result of altered synthesis and/or degradation; or altered trace amine load due to microbial action, diet, or altered trace amine transport properties. Such altered levels can then result in changes in chemotaxis and/or activation of leukocytes, and/or TA-induced changes in the ability of members of the microbiota to adhere to and penetrate the epithelial cell layer. Furthermore, in situations with primary dysbiosis, alterations of trace amine production by the constitutive microbiota could easily manifest, which may lead to loss of homeostasis of leukocyte chemotaxis and activation and/or increased sensitivity to foods containing trace amines or their precursors.

### A Role for TAARs in Immune Disorders of Other Organ Systems

In addition to a putative role for TAARs in gastrointestinal immune system dysfunction, coordination between the host immune system, epithelial cells, and the constitutive microbiota needs to occur and be tightly controlled in many other situations. In particular, a similar complex trilateral communication network is present in the pulmonary and urogenital systems, and a similar role for the trace aminergic system could occur in each of these. Furthermore, a role for dysregulated immune systems in neurologic and psychiatric disorders is becoming increasingly recognized. With few studies available, firm links in each of these systems requires a great deal more foundational work.

#### The Pulmonary System

In recent years, there has been a growing appreciation of possible links between pulmonary hypersensitivity disorders such as environmentally induced asthma, and dysbiosis (Dickson and Huffnagle, 2015; Hauptmann and Schaible, 2016), including gastrointestinal dysbiosis (Samuelson et al., 2015; Zhang I. et al., 2017). We are unaware of any studies directly implicating an altered trace amine metabolome with lung inflammation at this point in time, but the fact that all known endogenous TAAR ligands are highly volatile and present in a wide range of environmental contexts including food and animal urine and feces, while their receptors appear to play a role in regulating leukocyte chemotaxis and activation, suggests a putative link that warrants consideration. It is also worth noting that polymorphism of TAAR6 has been linked to corticosterone responsiveness in asthma patients (Chang et al., 2015). Corticosterone is not known to be a ligand for any TAAR, suggesting that TAAR6 may be contributing in an indirect manner.

#### The Urogenital Tract

Trimethylamine is sexually dimorphic in mice (Gavaghan McKee et al., 2006), and urinary levels are also reported to be increased in humans during menstruation (Mitchell and Smith, 2010) possibly due to a sex hormone-induced regulation of its degradation (Coecke et al., 1998; Shimizu et al., 2007). The socially stigmatizing odor associated with vaginal dysbiosis is largely a function of increased TMA levels (Fennema et al., 2016; Zhang and Davies, 2016). The production of putrescine, cadaverine, and TYR have been identified as important virulence factors in the progression of the dysbiotic environment (Nelson et al., 2015) and may underlie the increased incidence of bacterial vaginosis among smokers (Nelson et al., 2018).

#### The Nervous System

An inflammatory component to, at least, some neurological disorders is now recognized, and trace amines are synthesized in neurons, and then immediately released to the extra-neuronal environment by diffusion mediated events. Such released trace amines may recruit and activate leukocytes if the homeostatic equilibrium between synthesis and degradation is lost. While this is merely theoretical at this time, polymorphism of TAAR1 has been linked to fibromyalgia sensitivity (Smith et al., 2012), and fibromyalgia may involve an immune system component.

##### The central nervous system in IBD

Estimates suggest that upward of 33% of IBD patients report coincidence of non-gastrointestinal symptoms (Casella et al., 2014; Dolapcioglu and Dolapcioglu, 2015), and such symptoms may even precede gastrointestinal symptomatology. While neuropsychiatric complications are rare, IBD patients do show an increased risk of cerebrovascular disorders such as stroke (Dolapcioglu and Dolapcioglu, 2015; Nemati et al., 2017). IBD is also associated with upto a five-fold increase in demyelinating disorders such as multiple sclerosis that is independent of immunosuppressant therapy, and which may be related to a generalized loss of T lymphocyte homeostasis (Casella et al., 2014; Dolapcioglu and Dolapcioglu, 2015). Even though the role of TAARs have not been investigated with respect to demyelination, TAARs may be a future novel target for control of neuroinflammation given the increasing evidence for a putative role in regulating immune cell function and TAARs expression throughout the brain.

##### Inflammation in central nervous system disorders

There is an increasing interest in the “gut–brain axis,” with signals originating in the gastrointestinal tract, including from the constitutive microbiota, now accepted as regulating central nervous system function (Fung et al., 2017; Tremlett et al., 2017). Likewise, the association of low-grade inflammation with both increased risk of neuropsychiatric disorders and poor treatment outcomes is increasingly recognized (Osimo et al., 2018). Such inflammation can be associated with both the central and peripheral nervous systems and non-neural tissues such as epithelial cells (Chrysohoou et al., 2018; Nguyen et al., 2018), and has been identified as a novel therapeutic target (Osimo et al., 2018). Schizophrenia and bipolar disorder have both been reported to be associated with an increased prevalence of gastrointestinal disturbances, including increased inflammation and a dysfunctional epithelial barrier layer (Nguyen et al., 2018). Interestingly, a decrease in the constitutive microbiota diversity may also be associated with psychiatric disorders (Nguyen et al., 2018).

TAAR1 ligands are already in active development as treatments for neuropsychiatric disorders (Berry et al., 2017), and their potential to also modulate hyperactive immune function could offer an additional benefit. Of particular note is the spectrum of syndromes associated with chromosome 22q11.2 deletion that includes altered T lymphocyte differentiation resulting in hyper-reactive responses to environmental stimuli, and a marked increased risk of schizophrenia (Vergaelen et al., 2018). One of the genes located to 22q11.2 is catechol-*O*-methyltransferase, which catalyzes the conversion of dopamine and norepinephrine to 3-methoxytyramine and normetanephrine, both of which are potent TAAR1 agonists. As such chromosome 22q11.2 deletion likely leads to a deficit in basal TAAR1 signaling.

The role of inflammation and the constitutive microbiota in neuropsychiatric indications is an emerging area. One specific aspect that is unresolved is whether neuroinflammation results from infiltration of peripheral leukocytes in to the central nervous system due to a dysfunctional blood–brain barrier, activation of the central immune cells such as microglia, or a combination of both. With highly selective TAAR ligands becoming established as a way to normalize central neuronal function, the putative ability of the same compounds to normalize factors that regulate inflammatory effects would be a considerable advantage over existing therapeutics. As described in preceding sections, there is growing evidence that TAARs also play a role in regulating endothelial cell barrier functions and inflammatory responses in the gastrointestinal system, and possibly also in the placenta (Stavrou et al., 2018). Whether such effects also occur within the central nervous system is a key area for future systematic investigation. As far as we are aware, the TAAR expression profile of microglia and endothelial cells of the blood–brain barrier have not yet been examined.

## Concluding Statements

The trace aminergic system is an emerging target for pharmacotherapy that is garnering increasing interest. We propose that in addition to neurological/psychiatric and metabolic disorders, there is increasing evidence to support a putative role in environmental hypersensitivity immune system disorders, in particular IBD, and that a systematic investigation of the role of TAARs and their ligands in leukocyte homeostasis is warranted. We believe that such a putative role has an evolutionary basis. TAAR1, while evolutionarily the oldest family member, is the only isoform that is not involved in olfaction. This suggests that either this function has been lost in all species from the cartilaginous fish upward, or that co-opting TAARs to the olfactory epithelium following a gene duplication event offered an evolutionary advantage. Given that TAAR1 is under strong negative selection pressures, and that the second oldest family member, TAAR4, shares ligand selectivity with TAAR1, we believe this latter option is the most likely. The ability to detect compounds in the environment that can also trigger immune responses, and where that detection induces an innate avoidance response (as is known to occur), would offer a considerable advantage in protecting the organism from ingesting microbial contaminated foods that could be potentially injurious or even fatal. The fact that these compounds are also excreted in urine and feces due to intestinal microbiota metabolism of ingested meat, offers an additional advantage of triggering avoidance of areas associated with potential predators. It is also interesting to note that many aquatic species show an innate avoidance response to parasites and parasite-infected conspecifics, a response that is almost indistinguishable from their avoidance of predators. The chemical basis of this response is unknown, and TAARs could represent a new possibility for the molecular basis of such responses.

In conclusion, there is growing evidence that the trace aminergic system plays a role in the complex tripartite communication web between the epithelial cells that form a barrier between the host and the environment, host immune cells, and the constitutive microbiota, and that loss of trace amine homeostasis can be associated with immune system hyper-reactivity toward environmental cues.

## Author Contributions

Both authors contributed to the conception and generation of this hypothesis, wrote sections of the manuscript, contributed to manuscript revision, and read and approved the submitted version.

## Conflict of Interest Statement

The authors declare that the research was conducted in the absence of any commercial or financial relationships that could be construed as a potential conflict of interest.

## References

[B1] Adriaenssens A. Lam B. Y. Billing L. Skeffington K. Sewing S. Reimann F. (2015). A Transcriptome-led exploration of molecular mechanisms regulating somatostatin-producing D-cells in the gastric epithelium. *Endocrinology* 156 3924–3936. 10.1210/en.2015-1301 26241122 PMC4606756

[B2] Alexander S. P. Kelly E. Marrion N. V. Peters J. A. Faccenda E. Harding S. D. (2017). The concise guide to pharmacology. *Br. J. Pharmacol.* 174 S1–S446. 10.1111/bph.13882 29055035 PMC5650669

[B3] An D. Chen Z. Zheng J. Chen S. Wang L. Huang Z. (2015). Determination of biogenic amines in oysters by capillary electrophoresis coupled with electrochemiluminescence. *Food Chem.* 168 1–6. 10.1016/j.foodchem.2014.07.019 25172675

[B4] Babusyte A. Kotthoff M. Fiedler J. Krautwurst D. (2013). Biogenic amines activate blood leukocytes via trace amine-associated receptors TAAR1 and TAAR2. *J. Leukoc. Biol.* 93 387–394. 10.1189/jlb.0912433 23315425

[B5] Bamias G. Clark D. J. Rivera-Nieves J. (2013). Leukocyte traffic blockade as a therapeutic strategy in inflammatory bowel disease. *Curr. Drug Targets* 14 1490–1500. 10.2174/13894501113149990158 23621509 PMC3779486

[B6] Barak L. S. Salahpour A. Zhang X. Masri B. Sotnikova T. D. Ramsey A. J. (2008). Pharmacological characterization of membrane-expressed human trace amine-associated receptor 1 (TAAR1) by a bioluminescence resonance energy transfer cAMP biosensor. *Mol. Pharmacol.* 74 585–594. 10.1124/mol.108.048884 18524885 PMC3766527

[B7] Berry M. D. (2004). Mammalian central nervous system trace amines. Pharmacologic amphetamines, physiologic neuromodulators. *J. Neurochem.* 90 257–271. 10.1111/j.1471-4159.2004.02501.x 15228583

[B8] Berry M. D. Gainetdinov R. R. Hoener M. C. Shahid M. (2017). Pharmacology of human trace amine-associated receptors: therapeutic opportunities and challenges. *Pharmacol. Ther.* 180 161–180. 10.1016/j.pharmthera.2017.07.002 28723415

[B9] Berry M. D. Hart S. Pryor A. R. Hunter S. Gardiner D. (2016). Pharmacological characterization of a high-affinity p-tyramine transporter in rat brain synaptosomes. *Sci. Rep.* 6:38006. 10.1038/srep38006 27901065 PMC5128819

[B10] Berry M. D. Shitut M. R. Almousa A. Alcorn J. Tomberli B. (2013). Membrane permeability of trace amines: evidence for a regulated, activity-dependent, nonexocytotic, synaptic release. *Synapse* 67 656–667. 10.1002/syn.21670 23564683

[B11] Biji K. B. Ravishankar C. N. Venkateswarlu R. Mohan C. O. Gopal T. K. (2016). Biogenic amines in seafood: a review. *J. Food Sci. Technol.* 53 2210–2218. 10.1007/s13197-016-2224-x 27407186 PMC4921096

[B12] Bly M. (2005). Examination of the trace amine-associated receptor 2 (TAAR2). *Schizophr. Res.* 80 367–368. 10.1016/j.schres.2005.06.003 15993565

[B13] Borowsky B. Adham N. Jones K. A. Raddatz R. Artymyshyn R. Ogozalek K. L. (2001). Trace amines: identification of a family of mammalian G protein-coupled receptors. *Proc. Natl. Acad. Sci. U.S.A.* 98 8966–8971. 10.1073/pnas.151105198 11459929 PMC55357

[B14] Bradaia A. Trube G. Stalder H. Norcross R. D. Ozmen L. Wettstein J. G. (2009). The selective antagonist EPPTB reveals TAAR1-mediated regulatory mechanisms in dopaminergic neurons of the mesolimbic system. *Proc. Natl. Acad. Sci. U.S.A.* 106 20081–20086. 10.1073/pnas.0906522106 19892733 PMC2785295

[B15] Bunzow J. R. Sonders M. S. Arttamangkul S. Harrison L. M. Zhang G. Quigley D. I. (2001). Amphetamine, 3,4-methylenedioxymethamphetamine, lysergic acid diethylamide, and metabolites of the catecholamine neurotransmitters are agonists of a rat trace amine receptor. *Mol. Pharmacol.* 60 1181–1188. 10.1124/mol.60.6.1181 11723224

[B16] Burczynski M. E. Peterson R. L. Twine N. C. Zuberek K. A. Brodeur B. J. Casciotti L. (2006). Molecular classification of Crohn’s disease and ulcerative colitis patients using transcriptional profiles in peripheral blood mononuclear cells. *J. Mol. Diagn.* 8 51–61. 10.2353/jmoldx.2006.050079 16436634 PMC1867573

[B17] Casella G. Tontini G. E. Bassotti G. Pastorelli L. Villanacci V. Spina L. (2014). Neurological disorders and inflammatory bowel diseases. *World J. Gastroenterol.* 20 8764–8782. 10.3748/wjg.v20.i27.8764 25083051 PMC4112885

[B18] Chang H. S. Heo J. S. Shin S. W. Bae D. J. Song H. J. Jun J. A. (2015). Association between TAAR6 polymorphisms and airway responsiveness to inhaled corticosteroids in asthmatic patients. *Pharmacogenet. Genomics* 25 334–342. 10.1097/FPC.0000000000000141 25919112

[B19] Chiellini G. Erba P. Carnicelli V. Manfredi C. Frascarelli S. Ghelardoni S. (2012). Distribution of exogenous [125I]-3-iodothyronamine in mouse in vivo: relationship with trace amine-associated receptors. *J. Endocrinol.* 213 223–230. 10.1530/JOE-12-0055 22442117

[B20] Chiellini G. Frascarelli S. Ghelardoni S. Carnicelli V. Tobias S. C. DeBarber A. (2007). Cardiac effects of 3-iodothyronamine: a new aminergic system modulating cardiac function. *FASEB J.* 21 1597–1608. 10.1096/fj.06-7474com 17284482

[B21] Chrysohoou C. Kollia N. Tousoulis D. (2018). The link between depression and atherosclerosis through the pathways of inflammation and endothelium dysfunction. *Maturitas* 109 1–5. 10.1016/j.maturitas.2017.12.001 29452775

[B22] Churcher A. M. Hubbard P. C. Marques J. P. Canario A. V. Huertas M. (2015). Deep sequencing of the olfactory epithelium reveals specific chemosensory receptors are expressed at sexual maturity in the European eel Anguilla anguilla. *Mol. Ecol.* 24 822–834. 10.1111/mec.13065 25580852

[B23] Cisneros I. E. Ghorpade A. (2014). Methamphetamine and HIV-1-induced neurotoxicity: Role of trace amine associated receptor 1 cAMP signaling in astrocytes. *Neuropharmacology* 85C 499–507. 10.1016/j.neuropharm.2014.06.011 24950453 PMC4315503

[B24] Coecke S. Debast G. Phillips I. R. Vercruysse A. Shephard E. A. Rogiers V. (1998). Hormonal regulation of microsomal flavin-containing monooxygenase activity by sex steroids and growth hormone in co-cultured adult male rat hepatocytes. *Biochem. Pharmacol.* 56 1047–1051. 10.1016/S0006-2952(98)00104-X 9776317

[B25] Coutts R. T. Baker G. B. Pasutto F. M. (1986). Foodstuffs as sources of psychoactive amines and their precursors: content, significance and identification. *Adv. Drug Res.* 15 169–232.

[B26] Craciun S. Balskus E. P. (2012). Microbial conversion of choline to trimethylamine requires a glycyl radical enzyme. *Proc. Natl. Acad. Sci. U.S.A.* 109 21307–21312. 10.1073/pnas.1215689109 23151509 PMC3535645

[B27] D’Andrea G. D’Arrigo A. Facchinetti F. Del Giudice E. Colavito D. Bernardini D. (2012). Octopamine, unlike other trace amines, inhibits responses of astroglia-enriched cultures to lipopolysaccharide via a beta-adrenoreceptor-mediated mechanism. *Neurosci. Lett.* 517 36–40. 10.1016/j.neulet.2012.04.013 22507691

[B28] D’Andrea G. Terrazzino S. Fortin D. Farruggio A. Rinaldi L. Leon A. (2003). HPLC electrochemical detection of trace amines in human plasma and platelets and expression of mRNA transcripts of trace amine receptors in circulating leukocytes. *Neurosci. Lett.* 346 89–92. 10.1016/S0304-3940(03)00573-1 12850555

[B29] Dewan A. Pacifico R. Zhan R. Rinberg D. Bozza T. (2013). Non-redundant coding of aversive odours in the main olfactory pathway. *Nature* 497 486–489. 10.1038/nature12114 23624375 PMC3663888

[B30] Dickson R. P. Huffnagle G. B. (2015). The Lung Microbiome: New Principles for Respiratory Bacteriology in Health and Disease. *PLoS Pathog.* 11:e1004923. 10.1371/journal.ppat.1004923 26158874 PMC4497592

[B31] Dinter J. Muhlhaus J. Wienchol C. L. Yi C. X. Nurnberg D. Morin S. (2015). Inverse agonistic action of 3-iodothyronamine at the human trace amine-associated receptor 5. *PLoS One* 10:e0117774. 10.1371/journal.pone.0117774 25706283 PMC4382497

[B32] Dolapcioglu C. Dolapcioglu H. (2015). Structural brain lesions in inflammatory bowel disease. *World J. Gastrointest. Pathophysiol.* 6 124–130. 10.4291/wjgp.v6.i4.124 26600970 PMC4644876

[B33] Duan J. Martinez M. Sanders A. R. Hou C. Saitou N. Kitano T. (2004). Polymorphisms in the trace amine receptor 4 (TRAR4) gene on chromosome 6q23.2 are associated with susceptibility to schizophrenia. *Am. J. Hum. Genet.* 75 624–638. 10.1086/424887 15329799 PMC1182049

[B34] Durden D. A. Philips S. R. (1980). Kinetic measurements of the turnover rates of phenylethylamine and tryptamine in vivo in the rat brain. *J. Neurochem.* 34 1725–1732. 10.1111/j.1471-4159.1980.tb11267.x 7381498

[B35] Dyck L. E. (1989). Release of some endogenous trace amines from rat striatal slices in the presence and absence of a monoamine-oxidase inhibitor. *Life Sci.* 44 1149–1156. 10.1016/0024-3205(89)90309-3 2716465

[B36] Espinoza S. Ghisi V. Emanuele M. Leo D. Sukhanov I. Sotnikova T. D. (2015). Postsynaptic D2 dopamine receptor supersensitivity in the striatum of mice lacking TAAR1. *Neuropharmacology* 93 308–313. 10.1016/j.neuropharm.2015.02.010 25721394

[B37] Espinoza S. Salahpour A. Masri B. Sotnikova T. D. Messa M. Barak L. S. (2011). Functional interaction between trace amine-associated receptor 1 and dopamine D2 receptor. *Mol. Pharmacol.* 80 416–425. 10.1124/mol.111.073304 21670104 PMC3164335

[B38] Eyun S. I. Moriyama H. Hoffmann F. G. Moriyama E. N. (2016). Molecular Evolution and Functional Divergence of Trace Amine-Associated Receptors. *PLoS One* 11:e0151023. 10.1371/journal.pone.0151023 26963722 PMC4786312

[B39] Fatsini E. Bautista R. Manchado M. Duncan N. J. (2016). Transcriptomic profiles of the upper olfactory rosette in cultured and wild Senegalese sole (Solea senegalensis) males. *Comp. Biochem. Physiol. D Genomics Proteomics* 20 125–135. 10.1016/j.cbd.2016.09.001 27689822

[B40] Fennema D. Phillips I. R. Shephard E. A. (2016). Trimethylamine and trimethylamine N-oxide, a flavin-containing monooxygenase 3 (FMO3)-mediated host-microbiome metabolic axis implicated in health and disease. *Drug Metab. Dispos.* 44 1839–1850. 10.1124/dmd.116.070615 27190056 PMC5074467

[B41] Fernandez de Palencia P. Fernandez M. Mohedano M. L. Ladero V. Quevedo C. Alvarez M. A. (2011). Role of tyramine synthesis by food-borne Enterococcus durans in adaptation to the gastrointestinal tract environment. *Appl. Environ. Microbiol.* 77 699–702. 10.1128/AEM.01411-10 21097601 PMC3020552

[B42] Ferrero D. M. Lemon J. K. Fluegge D. Pashkovski S. L. Korzan W. J. Datta S. R. (2011). Detection and avoidance of a carnivore odor by prey. *Proc. Natl. Acad. Sci. U.S.A.* 108 11235–11240. 10.1073/pnas.1103317108 21690383 PMC3131382

[B43] Ferrero D. M. Wacker D. Roque M. A. Baldwin M. W. Stevens R. C. Liberles S. D. (2012). Agonists for 13 trace amine-associated receptors provide insight into the molecular basis of odor selectivity. *ACS Chem. Biol.* 7 1184–1189. 10.1021/cb300111e 22545963 PMC3401279

[B44] Fowler S. Kletzl H. Finel M. Manevski N. Schmid P. Tuerck D. (2015). A UGT2B10 splicing polymorphism common in african populations may greatly increase drug exposure. *J. Pharmacol. Exp. Ther.* 352 358–367. 10.1124/jpet.114.220194 25503386

[B45] Frank D. N. St Amand A. L. Feldman R. A. Boedeker E. C. Harpaz N. Pace N. R. (2007). Molecular-phylogenetic characterization of microbial community imbalances in human inflammatory bowel diseases. *Proc. Natl. Acad. Sci. U.S.A.* 104 13780–13785. 10.1073/pnas.0706625104 17699621 PMC1959459

[B46] Fung T. C. Olson C. A. Hsiao E. Y. (2017). Interactions between the microbiota, immune and nervous systems in health and disease. *Nat. Neurosci.* 20 145–155. 10.1038/nn.4476 28092661 PMC6960010

[B47] Gardini F. Ozogul Y. Suzzi G. Tabanelli G. Ozogul F. (2016). Technological factors affecting biogenic amine content in foods: a review. *Front. Microbiol.* 7:1218. 10.3389/fmicb.2016.01218 27570519 PMC4982241

[B48] Gavaghan McKee C. L. Wilson I. D. Nicholson J. K. (2006). Metabolic phenotyping of nude and normal (Alpk:ApfCD C57BL10J) mice. *J. Proteome Res.* 5 378–384. 10.1021/pr050255h 16457604

[B49] Gliem S. Schild D. Manzini I. (2009). Highly specific responses to amine odorants of individual olfactory receptor neurons in situ. *Eur. J. Neurosci.* 29 2315–2326. 10.1111/j.1460-9568.2009.06778.x 19490026

[B50] Gozal E. A. O’Neill B. E. Sawchuk M. A. Zhu H. Halder M. Chou C. C. (2014). Anatomical and functional evidence for trace amines as unique modulators of locomotor function in the mammalian spinal cord. *Front. Neural Circuits* 8:134. 10.3389/fncir.2014.00134 25426030 PMC4224135

[B51] Gurevich V. V. Gurevich E. V. (2014). Arrestin makes T cells stop and become active. *EMBO J.* 33 531–533. 10.1002/embj.201387724 24502974 PMC3989646

[B52] Harmeier A. Obermueller S. Meyer C. A. Revel F. G. Buchy D. Chaboz S. (2015). Trace amine-associated receptor 1 activation silences GSK3beta signaling of TAAR1 and D2R heteromers. *Eur. Neuropsychopharmacol.* 25 2049–2061. 10.1016/j.euroneuro.2015.08.011 26372541

[B53] Hashiguchi Y. Nishida M. (2007). Evolution of trace amine associated receptor (TAAR) gene family in vertebrates: lineage-specific expansions and degradations of a second class of vertebrate chemosensory receptors expressed in the olfactory epithelium. *Mol. Biol. Evol.* 24 2099–2107. 10.1093/molbev/msm140 17634392

[B54] Hauptmann M. Schaible U. E. (2016). Linking microbiota and respiratory disease. *FEBS Lett.* 590 3721–3738. 10.1002/1873-3468.12421 27637588

[B55] Henry D. P. Russell W. L. Clemens J. A. Plebus L. A. (1988). “Phenylethylamine and p-tyramine in the extracellular space of the rat brain: quantification using a new radioenzymatic assay and in situ microdialysis,” in *Trace Amines; Comparative and Clinical Neurobiology* eds Boulton A. A. Juorio A. V. Downer R. G. H. (Clifton, NJ: Humana Press) 239–250.

[B56] Horowitz L. F. Saraiva L. R. Kuang D. Yoon K. H. Buck L. B. (2014). Olfactory receptor patterning in a higher primate. *J. Neurosci.* 34 12241–12252. 10.1523/JNEUROSCI.1779-14.2014 25209267 PMC4160765

[B57] Hussain A. Saraiva L. R. Ferrero D. M. Ahuja G. Krishna V. S. Liberles S. D. (2013). High-affinity olfactory receptor for the death-associated odor cadaverine. *Proc. Natl. Acad. Sci. U.S.A.* 110 19579–19584. 10.1073/pnas.1318596110 24218586 PMC3845148

[B58] Hussain A. Saraiva L. R. Korsching S. I. (2009). Positive Darwinian selection and the birth of an olfactory receptor clade in teleosts. *Proc. Natl. Acad. Sci. U.S.A.* 106 4313–4318. 10.1073/pnas.0803229106 19237578 PMC2657432

[B59] Irsfeld M. Spadafore M. Pruss B. M. (2013). β-Phenylethylamine, a small molecule with a large impact. *Webmedcentral* 4:4409. PMC3904499 24482732

[B60] Ito J. Ito M. Nambu H. Fujikawa T. Tanaka K. Iwaasa H. (2009). Anatomical and histological profiling of orphan G-protein-coupled receptor expression in gastrointestinal tract of C57BL/6J mice. *Cell Tissue Res.* 338 257–269. 10.1007/s00441-009-0859-x 19763624

[B61] Izquierdo C. Gomez-Tamayo J. C. Nebel J. C. Pardo L. Gonzalez A. (2018). Identifying human diamine sensors for death related putrescine and cadaverine molecules. *PLoS Comput. Biol.* 14:e1005945. 10.1371/journal.pcbi.1005945 29324768 PMC5783396

[B62] Jacobs J. P. Goudarzi M. Singh N. Tong M. McHardy I. H. Ruegger P. (2016). A disease-associated microbial and metabolomics state in relatives of pediatric inflammatory bowel disease patients. *Cell Mol. Gastroenterol. Hepatol.* 2 750–766. 10.1016/j.jcmgh.2016.06.004 28174747 PMC5247316

[B63] Jansson J. Willing B. Lucio M. Fekete A. Dicksved J. Halfvarson J. (2009). Metabolomics reveals metabolic biomarkers of Crohn’s disease. *PLoS One* 4:e6386. 10.1371/journal.pone.0006386 19636438 PMC2713417

[B64] Jiang D. Xie T. Liang J. Noble P. W. (2013). beta-Arrestins in the immune system. *Prog. Mol. Biol. Transl. Sci.* 118 359–393. 10.1016/B978-0-12-394440-5.00014-0 23764061 PMC4467910

[B65] Jostins L. Ripke S. Weersma R. K. Duerr R. H. McGovern D. P. Hui K. Y. (2012). Host-microbe interactions have shaped the genetic architecture of inflammatory bowel disease. *Nature* 491 119–124. 10.1038/nature11582 23128233 PMC3491803

[B66] Juorio A. V. Greenshaw A. J. Wishart T. B. (1988). Reciprocal changes in striatal dopamine and b-phenylethylamine induced by reserpine in the presence of monoamine oxidase inhibitors. *Naunyn Schmiedebergs Arch. Pharmacol.* 338 644–648. 10.1007/BF00165628 3149722

[B67] Kalnins G. Kuka J. Grinberga S. Makrecka-Kuka M. Liepinsh E. Dambrova M. (2015). Structure and function of CutC choline lyase from human microbiota bacterium *Klebsiella pneumoniae*. *J. Biol. Chem.* 290 21732–21740. 10.1074/jbc.M115.670471 26187464 PMC4571895

[B68] Kanageswaran N. Demond M. Nagel M. Schreiner B. S. Baumgart S. Scholz P. (2015). Deep sequencing of the murine olfactory receptor neuron transcriptome. *PLoS One* 10:e0113170. 10.1371/journal.pone.0113170 25590618 PMC4295871

[B69] Kidd M. Modlin I. M. Gustafsson B. I. Drozdov I. Hauso O. Pfragner R. (2008). Luminal regulation of normal and neoplastic human EC cell serotonin release is mediated by bile salts, amines, tastants, and olfactants. *Am. J. Physiol. Gastrointest. Liver Physiol.* 295 G260–G272. 10.1152/ajpgi.00056.2008 18556422

[B70] Kostic A. D. Xavier R. J. Gevers D. (2014). The microbiome in inflammatory bowel disease: current status and the future ahead. *Gastroenterology* 146 1489–1499. 10.1053/j.gastro.2014.02.009 24560869 PMC4034132

[B71] Lane E. R. Zisman T. L. Suskind D. L. (2017). The microbiota in inflammatory bowel disease: current and therapeutic insights. *J. Inflamm. Res.* 10 63–73. 10.2147/JIR.S116088 28652796 PMC5473501

[B72] Lang D. H. Yeung C. K. Peter R. M. Ibarra C. Gasser R. Itagaki K. (1998). Isoform specificity of trimethylamine *N*-oxygenation by human flavin-containing monooxygenase (FMO) and P450 enzymes: Selective catalysis by fmo3. *Biochem. Pharmacol.* 56 1005–1012. 10.1016/S0006-2952(98)00218-4 9776311

[B73] Lauweryns J. M. Van Ranst L. (1988). Immunocytochemical localization of aromatic L-amino acid decarboxylase in human, rat, and mouse bronchopulmonary and gastrointestinal endocrine cells. *J. Histochem. Cytochem.* 36 1181–1186. 10.1177/36.9.2900264 2900264

[B74] Lee Y. K. Mazmanian S. K. (2010). Has the microbiota played a critical role in the evolution of the adaptive immune system? *Science* 330 1768–1773. 10.1126/science.1195568 21205662 PMC3159383

[B75] Lewis J. D. Chen E. Z. Baldassano R. N. Otley A. R. Griffiths A. M. Lee D. (2015). Inflammation, antibiotics, and diet as environmental stressors of the gut microbiome in pediatric Crohn’s disease. *Cell Host Microbe* 18 489–500. 10.1016/j.chom.2015.09.008 26468751 PMC4633303

[B76] Li Q. Korzan W. J. Ferrero D. M. Chang R. B. Roy D. S. Buchi M. (2013). Synchronous evolution of an odor biosynthesis pathway and behavioral response. *Curr. Biol.* 23 11–20. 10.1016/j.cub.2012.10.047 23177478 PMC3543494

[B77] Li Q. Tachie-Baffour Y. Liu Z. Baldwin M. W. Kruse A. C. Liberles S. D. (2015). Non-classical amine recognition evolved in a large clade of olfactory receptors. *eLife* 4:e10441. 10.7554/eLife.10441 26519734 PMC4695389

[B78] Liberles S. D. (2015). Trace amine-associated receptors: ligands, neural circuits, and behaviors. *Curr. Opin. Neurobiol.* 34 1–7. 10.1016/j.conb.2015.01.001 25616211 PMC4508243

[B79] Liberles S. D. Buck L. B. (2006). A second class of chemosensory receptors in the olfactory epithelium. *Nature* 442 645–650. 10.1038/nature05066 16878137

[B80] Lindemann L. Ebeling M. Kratochwil N. A. Bunzow J. R. Grandy D. K. Hoener M. C. (2005). Trace amine-associated receptors form structurally and functionally distinct subfamilies of novel G protein-coupled receptors. *Genomics* 85 372–385. 10.1016/j.ygeno.2004.11.010 15718104

[B81] Lindemann L. Meyer C. A. Jeanneau K. Bradaia A. Ozmen L. Bluethmann H. (2008). Trace amine-associated receptor 1 modulates dopaminergic activity. *J. Pharmacol. Exp. Ther.* 324 948–956. 10.1124/jpet.107.132647 18083911

[B82] Linnoila R. I. Gazdar A. F. Funa K. Becker K. L. (1993). Long-term selective culture of hamster pulmonary endocrine cells. *Anat. Rec.* 236 231–240. 10.1002/ar.1092360128 8389531

[B83] Luqman A. Nega M. Nguyen M. T. Ebner P. Gotz F. (2018). SadA-expressing staphylococci in the human gut show increased cell adherence and internalization. *Cell Rep.* 22 535–545. 10.1016/j.celrep.2017.12.058 29320746

[B84] Magro F. Cunha E. Araujo F. Meireles E. Pereira P. Dinis-Ribeiro M. (2006). Dopamine D2 receptor polymorphisms in inflammatory bowel disease and the refractory response to treatment. *Dig. Dis. Sci.* 51 2039–2044. 10.1007/s10620-006-9168-3 16977509

[B85] Marchesi J. R. Holmes E. Khan F. Kochhar S. Scanlan P. Shanahan F. (2007). Rapid and noninvasive metabonomic characterization of inflammatory bowel disease. *J. Proteome Res.* 6 546–551. 10.1021/pr060470d 17269711

[B86] Marcobal A. de las Rivas B. Munoz R. (2006). First genetic characterization of a bacterial beta-phenylethylamine biosynthetic enzyme in Enterococcus faecium RM58. *FEMS Microbiol. Lett.* 258 144–149. 10.1111/j.1574-6968.2006.00206.x 16630269

[B87] Martini E. Krug S. M. Siegmund B. Neurath M. F. Becker C. (2017). Mend your fences: the epithelial barrier and its relationship with mucosal immunity in inflammatory bowel disease. *Cell Mol. Gastroenterol. Hepatol.* 4 33–46. 10.1016/j.jcmgh.2017.03.007 28560287 PMC5439240

[B88] Miller-Fleming L. Olin-Sandoval V. Campbell K. Ralser M. (2015). Remaining mysteries of molecular biology: the role of polyamines in the cell. *J. Mol. Biol.* 427 3389–3406. 10.1016/j.jmb.2015.06.020 26156863

[B89] Mitchell S. C. Smith R. L. (2010). A physiological role for flavin-containing monooxygenase (FMO3) in humans? *Xenobiotica* 40 301–305. 10.3109/00498251003702753 20230247

[B90] Mitchell S. C. Smith R. L. (2016). Trimethylamine-the extracorporeal envoy. *Chem. Senses* 41 275–279. 10.1093/chemse/bjw001 26809486

[B91] Muller D. J. Chiesa A. Mandelli L. De Luca V. De Ronchi D. Jain U. (2010). Correlation of a set of gene variants, life events and personality features on adult ADHD severity. *J. Psychiatr. Res.* 44 598–604. 10.1016/j.jpsychires.2009.11.011 20006992

[B92] Nazimek J. Perini F. Capitao L. McKie S. Browning M. Harmer C. (2016). A phase I functional neuroimaging study of SEP-363856 in healthy volunteers with high or low stereotypy. *Neuropsychopharmacology* 41 S393–S394.

[B93] Nelson D. A. Tolbert M. D. Singh S. J. Bost K. L. (2007). Expression of neuronal trace amine-associated receptor (Taar) mRNAs in leukocytes. *J. Neuroimmunol.* 192 21–30. 10.1016/j.jneuroim.2007.08.006 17900709 PMC2189554

[B94] Nelson T. M. Borgogna J. C. Michalek R. D. Roberts D. W. Rath J. M. Glover E. D. (2018). Cigarette smoking is associated with an altered vaginal tract metabolomic profile. *Sci. Rep.* 8:852. 10.1038/s41598-017-14943-3 29339821 PMC5770521

[B95] Nelson T. M. Borgogna J. L. Brotman R. M. Ravel J. Walk S. T. Yeoman C. J. (2015). Vaginal biogenic amines: biomarkers of bacterial vaginosis or precursors to vaginal dysbiosis? *Front. Physiol.* 6:253 10.3389/fphys.2015.00253 PMC4586437 26483694

[B96] Nemati R. Mehdizadeh S. Salimipour H. Yaghoubi E. Alipour Z. Tabib S. M. (2017). Neurological manifestations related to Crohn’s disease: a boon for the workforce. *Gastroenterol. Rep.* 10.1093/gastro/gox034 PMC6688734 31413837

[B97] Nguyen T. T. Kosciolek T. Eyler L. T. Knight R. Jeste D. V. (2018). Overview and systematic review of studies of microbiome in schizophrenia and bipolar disorder. *J. Psychiatr. Res.* 99 50–61. 10.1016/j.jpsychires.2018.01.013 29407287 PMC5849533

[B98] Ohta H. Takebe Y. Murakami Y. Takahama Y. Morimura S. (2017). Tyramine and beta-phenylethylamine, from fermented food products, as agonists for the human trace amine-associated receptor 1 (hTAAR1) in the stomach. *Biosci. Biotechnol. Biochem.* 81 1002–1006. 10.1080/09168451.2016.1274640 28084165

[B99] Osimo E. F. Cardinal R. N. Jones P. B. Khandaker G. M. (2018). Prevalence and correlates of low-grade systemic inflammation in adult psychiatric inpatients: an electronic health record-based study. *Psychoneuroendocrinology* 91 226–234. 10.1016/j.psyneuen.2018.02.031 29544672 PMC5910056

[B100] Pacheco R. Contreras F. Zouali M. (2014). The dopaminergic system in autoimmune diseases. *Front. Immunol.* 5:117. 10.3389/fimmu.2014.00117 24711809 PMC3968755

[B101] Pae C. U. Drago A. Kim J. J. Patkar A. A. Jun T. Y. De Ronchi D. (2010). TAAR6 variations possibly associated with antidepressant response and suicidal behavior. *Psychiatry Res.* 180 20–24. 10.1016/j.psychres.2009.08.007 20493543

[B102] Pae C. U. Drago A. Kim J. J. Patkar A. A. Jun T. Y. Lee C. (2008). TAAR6 variation effect on clinic presentation and outcome in a sample of schizophrenic in-patients: an open label study. *Eur. Psychiatry* 23 390–395. 10.1016/j.eurpsy.2008.04.004 18583103

[B103] Papa I. Saliba D. Ponzoni M. Bustamante S. Canete P. F. Gonzalez-Figueroa P. (2017). TFH-derived dopamine accelerates productive synapses in germinal centres. *Nature* 547 318–323. 10.1038/nature23013 28700579 PMC5540173

[B104] Pegg A. E. (2016). Functions of polyamines in mammals. *J. Biol. Chem.* 291 14904–14912. 10.1074/jbc.R116.731661 27268251 PMC4946908

[B105] Pei Y. Asif-Malik A. Canales J. J. (2016). Trace amines and the trace amine-associated receptor 1: pharmacology, neurochemistry, and clinical implications. *Front. Neurosci.* 10:148. 10.3389/fnins.2016.00148 27092049 PMC4820462

[B106] Pessione E. Cirrincione S. (2016). Bioactive molecules released in food by lactic acid bacteria: encrypted peptides and biogenic amines. *Front. Microbiol.* 7:876. 10.3389/fmicb.2016.00876 27375596 PMC4899451

[B107] Raab S. Wang H. Uhles S. Cole N. Alvarez-Sanchez R. Kunnecke B. (2016). Incretin-like effects of small molecule trace amine-associated receptor 1 agonists. *Mol. Metab.* 5 47–56. 10.1016/j.molmet.2015.09.015 26844206 PMC4703809

[B108] Regard J. B. Sato I. T. Coughlin S. R. (2008). Anatomical profiling of G protein-coupled receptor expression. *Cell* 135 561–571. 10.1016/j.cell.2008.08.040 18984166 PMC2590943

[B109] Revel F. G. Moreau J. L. Pouzet B. Mory R. Bradaia A. Buchy D. (2013). A new perspective for schizophrenia: TAAR1 agonists reveal antipsychotic- and antidepressant-like activity, improve cognition and control body weight. *Mol. Psychiatry* 18 543–556. 10.1038/mp.2012.57 22641180

[B110] Salahpour A. Espinoza S. Masri B. Lam V. Barak L. S. Gainetdinov R. R. (2012). BRET biosensors to study GPCR biology, pharmacology, and signal transduction. *Front. Endocrinol.* 3:105 10.3389/fendo.2012.00105 PMC3430160 22952466

[B111] Samuelson D. R. Welsh D. A. Shellito J. E. (2015). Regulation of lung immunity and host defense by the intestinal microbiota. *Front. Microbiol.* 6:1085. 10.3389/fmicb.2015.01085 26500629 PMC4595839

[B112] Santoru M. L. Piras C. Murgia A. Palmas V. Camboni T. Liggi S. (2017). Cross sectional evaluation of the gut-microbiome metabolome axis in an Italian cohort of IBD patients. *Sci. Rep.* 7:9523. 10.1038/s41598-017-10034-5 28842640 PMC5573342

[B113] Santos P. S. Courtiol A. Heidel A. J. Honer O. P. Heckmann I. Nagy M. (2016). MHC-dependent mate choice is linked to a trace-amine-associated receptor gene in a mammal. *Sci. Rep.* 6:38490. 10.1038/srep38490 27941813 PMC5150237

[B114] Scanlan T. S. Suchland K. L. Hart M. E. Chiellini G. Huang Y. Kruzich P. J. (2004). 3-Iodothyronamine is an endogenous and rapid-acting derivative of thyroid hormone. *Nat. Med.* 10 638–642. 10.1038/nm1051 15146179

[B115] Shi X. Walter N. A. Harkness J. H. Neve K. A. Williams R. W. Lu L. (2016). Genetic polymorphisms affect mouse and human trace amine-associated receptor 1 function. *PLoS One* 11:e0152581. 10.1371/journal.pone.0152581 27031617 PMC4816557

[B116] Shimizu M. Cashman J. R. Yamazaki H. (2007). Transient trimethylaminuria related to menstruation. *BMC Med. Genet.* 8:2. 10.1186/1471-2350-8-2 17257434 PMC1790885

[B117] Sigall-Boneh R. Levine A. Lomer M. Wierdsma N. Allan P. Fiorino G. (2017). Research gaps in diet and nutrition in inflammatory bowel disease. A topical review by d-ecco working group [Dietitians of ECCO]. *J. Crohns Colitis* 11 1407–1419. 10.1093/ecco-jcc/jjx109 28961811

[B118] Simmler L. D. Buchy D. Chaboz S. Hoener M. C. Liechti M. E. (2016). In vitro characterization of psychoactive substances at rat, mouse, and human trace amine-associated receptor 1. *J. Pharmacol. Exp. Ther.* 357 134–144. 10.1124/jpet.115.229765 26791601

[B119] Smith S. B. Maixner D. W. Fillingim R. B. Slade G. Gracely R. H. Ambrose K. (2012). Large candidate gene association study reveals genetic risk factors and therapeutic targets for fibromyalgia. *Arthritis Rheum.* 64 584–593. 10.1002/art.33338 21905019 PMC3237946

[B120] Sotnikova T. D. Beaulieu J. M. Espinoza S. Masri B. Zhang X. Salahpour A. (2010). The dopamine metabolite 3-methoxytyramine is a neuromodulator. *PLoS One* 5:e13452. 10.1371/journal.pone.0013452 20976142 PMC2956650

[B121] Sriram U. Cenna J. M. Haldar B. Fernandes N. C. Razmpour R. Fan S. (2016). Methamphetamine induces trace amine-associated receptor 1 (TAAR1) expression in human T lymphocytes: role in immunomodulation. *J. Leukoc. Biol.* 99 213–223. 10.1189/jlb.4A0814-395RR 26302754 PMC4673484

[B122] Stavrou S. Gratz M. Tremmel E. Kuhn C. Hofmann S. Heidegger H. (2018). TAAR1 induces a disturbed GSK3beta phosphorylation in recurrent miscarriages through the ODC. *Endocr. Connect.* 7 372–384. 10.1530/EC-17-0272 29472377 PMC5825928

[B123] Syed A. S. Sansone A. Roner S. Bozorg Nia S. Manzini I. Korsching S. I. (2015). Different expression domains for two closely related amphibian TAARs generate a bimodal distribution similar to neuronal responses to amine odors. *Sci. Rep.* 5:13935. 10.1038/srep13935 26358883 PMC4566120

[B124] Taquet N. Philippe C. Reimund J.-M. Muller C. D. (2012). “Inflammatory bowel disease G-protein coupled receptors (GPCRs) expression profiling with microfluidic cards,” in *Crohn’s Disease* ed. Karoui S. (Rijeka: Intech open) 59–86.

[B125] Thomas S. Baumgart D. C. (2012). Targeting leukocyte migration and adhesion in Crohn’s disease and ulcerative colitis. *Inflammopharmacology* 20 1–18. 10.1007/s10787-011-0104-6 22205271

[B126] Toro-Funes N. Bosch-Fuste J. Latorre-Moratalla M. L. Veciana-Nogues M. T. Vidal-Carou M. C. (2015). Biologically active amines in fermented and non-fermented commercial soybean products from the Spanish market. *Food Chem.* 173 1119–1124. 10.1016/j.foodchem.2014.10.118 25466133

[B127] Tremlett H. Bauer K. C. Appel-Cresswell S. Finlay B. B. Waubant E. (2017). The gut microbiome in human neurological disease: a review. *Ann. Neurol.* 81 369–382. 10.1002/ana.24901 28220542

[B128] Vallender E. J. Xie Z. Westmoreland S. V. Miller G. M. (2010). Functional evolution of the trace amine associated receptors in mammals and the loss of TAAR1 in dogs. *BMC Evol. Biol.* 10:51. 10.1186/1471-2148-10-51 20167089 PMC2838891

[B129] Vanti W. B. Muglia P. Nguyen T. Cheng R. Kennedy J. L. George S. R. (2003). Discovery of a null mutation in a human trace amine receptor gene. *Genomics* 82 531–536. 10.1016/S0888-7543(03)00173-3 14559210

[B130] Vergaelen E. Schiweck C. Van Steeland K. Counotte J. Veling W. Swillen A. (2018). A pilot study on immuno-psychiatry in the 22q11.2 deletion syndrome: a role for Th17 cells in psychosis? *Brain Behav. Immun.* 70 88–95. 10.1016/j.bbi.2018.03.022 29567371 PMC6206432

[B131] Vieira-Coelho M. A. Soares-da-Silva P. (1993). Dopamine formation, from its immediate precursor 3,4-dihydroxyphenylalanine, along the rat digestive tract. *Fundam. Clin. Pharmacol.* 7 235–243. 10.1111/j.1472-8206.1993.tb00237.x 8370570

[B132] Vitale S. Strisciuglio C. Pisapia L. Miele E. Barba P. Vitale A. (2017). Cytokine production profile in intestinal mucosa of paediatric inflammatory bowel disease. *PLoS One* 12:e0182313. 10.1371/journal.pone.0182313 28797042 PMC5552230

[B133] Wallrabenstein I. Kuklan J. Weber L. Zborala S. Werner M. Altmuller J. (2013). Human trace amine-associated receptor TAAR5 can be activated by trimethylamine. *PLoS One* 8:e54950. 10.1371/journal.pone.0054950 23393561 PMC3564852

[B134] Wasik A. M. Millan M. J. Scanlan T. Barnes N. M. Gordon J. (2012). Evidence for functional trace amine associated receptor-1 in normal and malignant B cells. *Leuk. Res.* 36 245–249. 10.1016/j.leukres.2011.10.002 22036195 PMC5293406

[B135] Williams B. B. Van Benschoten A. H. Cimermancic P. Donia M. S. Zimmermann M. Taketani M. (2014). Discovery and characterization of gut microbiota decarboxylases that can produce the neurotransmitter tryptamine. *Cell Host Microbe* 16 495–503. 10.1016/j.chom.2014.09.001 25263219 PMC4260654

[B136] Wilson A. Teft W. A. Morse B. L. Choi Y. H. Woolsey S. DeGorter M. K. (2015). Trimethylamine-N-oxide: a novel biomarker for the identification of inflammatory bowel disease. *Dig. Dis. Sci.* 60 3620–3630. 10.1007/s10620-015-3797-3 26160437

[B137] Zeng L. X. Tao J. Liu H. L. Tan S. W. Yang Y. D. Peng X. J. (2015). beta-Arrestin2 encourages inflammation-induced epithelial apoptosis through ER stress/PUMA in colitis. *Mucosal Immunol* 8 683–695. 10.1038/mi.2014.104 25354317

[B138] Zhang I. Pletcher S. D. Goldberg A. N. Barker B. M. Cope E. K. (2017). Fungal microbiota in chronic airway inflammatory disease and emerging relationships with the host immune response. *Front. Microbiol.* 8:2477. 10.3389/fmicb.2017.02477 29312187 PMC5733051

[B139] Zhang J. Pacifico R. Cawley D. Feinstein P. Bozza T. (2013). Ultrasensitive detection of amines by a trace amine-associated receptor. *J. Neurosci.* 33 3228–3239. 10.1523/JNEUROSCI.4299-12.2013 23407976 PMC3711460

[B140] Zhang L. S. Davies S. S. (2016). Microbial metabolism of dietary components to bioactive metabolites: opportunities for new therapeutic interventions. *Genome Med.* 8:46. 10.1186/s13073-016-0296-x 27102537 PMC4840492

[B141] Zhang M. Sun K. J. Wu Y. J. Yang Y. Tso P. Wu Z. L. (2017). Interactions between intestinal microbiota and host immune response in inflammatory bowel disease. *Front. Immunol.* 8:942. 10.3389/fimmu.2017.00942. 28855901 PMC5558048

[B142] Zhu Y. Jameson E. Crosatti M. Schafer H. Rajakumar K. Bugg T. D. (2014). Carnitine metabolism to trimethylamine by an unusual Rieske-type oxygenase from human microbiota. *Proc. Natl. Acad. Sci. U.S.A.* 111 4268–4273. 10.1073/pnas.1316569111 24591617 PMC3964110

